# 

**Published:** 1814-02

**Authors:** 


### MONTHLY CATALOGUE OF MEDICAL BOOKS.

AN Account of a successful Method of treating Diseases of the Spine. By Thomas Baynton, of Bristol. 8vo. 5s. 6d.

Medico-Chirurgical Transactions, published by the Medical and Chirurgical Society of London. Vol. IV. 8vo. boards, 1l. 1s.

The Chemical Catechism, with Notes, &c. By Samuel Parkes, F.L.S. &c. The Sixth Edition, with Additions. 8vo. boards. 12s.

Facts and Observations relative to the Fever commonly called Puerperal. By John Armstrong, M.D. 8vo. boards. 8s. 6d.

Callow's Catalogue of Medical Books, for 1814; containing the most modern and approved Authors on Anatomy; Surgery, Medicine, Midwifery, Materia Medica, Chemistry, Pharmacy, Botany, Veterinary Medicine, &c. &c.

### *NOTICES TO CORRESPONDENTS.*

*Dr. Adams's paper did not arrive till the part of our Journal devoted to original communications had been printed. On the Doctor's authority we venture to suggest an erratum, which we have now no opportunity of examining with the MS.—Should not the first line of page* 8 *in our last Number, instead of* "not within," *be* "met with in?"

*We regret that the communication of* "*An Admirer of Truth and Justice,*" *in refutation of the article in our last Journal,* "*On the inefficacy of Antim. Tartar. in the cure of Ophthalmia,*" *was received too late for insertion.*

*Dr. R. C. Wilbraham, and our Exeter friend, are assured, that notwithstanding some insidious attempts to interfere with the establishment of this Work, our sale has not been greater for many months than during the past month; and that no periodical Medical Work has ever existed in the English Language which, judging from the numbers regularly sold, has been more extensively read at home and abroad than is this Journal.*

*Some accidental circumstances, with which it is unnecessary to trouble our readers, occasioned the errors of the press of which* Grammaticus *complains*

